# Entrustable Professional Activities in Residency Training: A Qualitative Study on Trainees’ Perceptions Using the Consolidated Framework for Implementation Research

**DOI:** 10.5334/pme.2076

**Published:** 2026-11-02

**Authors:** Fabienne Schwitz, Vera Bissig, Monika Brodmann Maeder, Eva K. Hennel

**Affiliations:** 1 Department of Cardiology, Inselspital, Bern University Hospital, University of Bern, Switzerland; 2 Swiss Institute for Medical Education (Schweizerisches Institut für ärztliche Weiter- und Fortbildung SIWF), Bern, Switzerland; 3 Medical Faculty, University of Bern, Switzerland; 4 Affiliated with the Institute for Medical Education, University of Bern, Switzerland

## Abstract

**Introduction:**

Entrustable Professional Activities (EPAs) represent an integral component of Competency-Based Medical Education (CBME). They provide a practical framework to assess a trainee’s readiness to perform clinical tasks with increasing independence. This study investigates trainees’ perceptions of EPAs, including benefits, obstacles, shifts in feedback culture, and changes in assumed responsibility using the Consolidated Framework for Implementation Research (CFIR) as a theoretical framework.

**Methods:**

We conducted a theory-informed qualitative study using semi-structured interviews with cardiology trainees in Switzerland to examine early implementation of Entrustable Professional Activities (EPAs). The study was informed by the Consolidated Framework for Implementation Research (CFIR). Data were analysed using the Framework Method, drawing on CFIR to inform the deductive analysis while identifying context-specific aspects of participants’ experiences inductively.

**Results:**

We identified ten themes, of which the most prominent were ‘Impact of time constraints for EPA assessment’, ‘Incorporating EPAs into workflows’, ‘Satisfaction with EPA App’, and ‘Enhancement of Feedback through EPAs’. Trainees perceived the use of EPAs as increasing feedback opportunities but reported that their educational value depended largely on time availability, workflow integration, and supervisor engagement.

**Discussion:**

Our findings suggest that the implementation and educational value of EPAs depend not only on the introduction of EPA-based assessments themselves but also on the interaction between structural conditions and social factors. Based on these findings, we formulated seven recommendations for training sites implementing EPAs. These are: Carry out assessments carefully, assess EPAs at handover, allocate time for assessments, begin assessments early in postgraduate medical education, provide all affected parties with thorough training, improve didactic competencies and share responsibility of assessments.

## Introduction

Entrustable Professional Activities (EPAs) represent an integral component of competency-based medical education (CBME) [1]. CBME de-emphasises time-based training, tailoring residency duration to trainees’ competency achievements [2]. EPAs link theory to clinical competence by entrusting trainees with increasing independence as they demonstrate competency [3]. As trainees develop skills, they earn supervisors’ trust, a crucial aspect in postgraduate medical education [4]. EPAs are assessed on five levels of independence to support and entrust trainees with appropriate responsibility.

Current literature on EPAs includes studies supporting the use of EPAs as a novel approach in postgraduate medical education [5,6,7], feasibility and evaluation studies [8,9,10,11] and studies seeking validity support for EPA-based assessments and entrustment-based decisions [12,13]. While the literature highlights EPAs as a promising approach in postgraduate medical education, there is little research on their implementation. This leaves significant gaps in understanding their application and how to effectively implement EPAs [14]. Previous studies have explored barriers and facilitators using implementation frameworks such as the Consolidated Framework for Implementation Research [15], and other work has also focused on the perspectives of the supervisors and curriculum leaders [16]. Hence, we evaluate the implementation process of an EPA-based curriculum with an interview-based, qualitative study employing CFIR and capturing the important perspective of trainees [17,18].

This study aimed to explore how cardiology trainees perceive the implementation of Entrustable Professional Activities (EPAs) in order to inform the use of EPAs in medical training. Our research question was:

“How do cardiology trainees perceive the implementation of EPAs in their training programs, with regard to benefits, obstacles, and changes in the feedback culture and responsibility?”

## Methods

### Study Design and Theoretical Framework

We chose a qualitative interview-based study design taking a pragmatic approach [19,20,21] and report the work following the O’Brien [22] standards for reporting qualitative research. The pragmatic approach is oriented toward addressing the research question through the use of methods that best capture relevant perspectives and generate actionable insights, acknowledging that knowledge is constructed through the interaction between researchers, participants, and the context of inquiry. We used the Consolidated Framework for Implementation Research (CFIR) [17] as a theoretical framework to plan the interviews and guide the analysis. In line with the pragmatic approach, CFIR was used as a flexible, sensitizing framework to support data analysis, rather than as a positivist model to test predefined implementation determinants. It enabled both deductive construct-based organisation of data and inductive identification of context-specific aspects of participants’ accounts.

The introduction of EPAs represents a substantial change in educational practice, requiring shifts in assessment culture, supervisory roles, and organisational processes. We therefore adopted the CFIR to guide our evaluation, as it provides a structured lens to examine how characteristics of the intervention, the inner and outer setting, individual actors, and implementation processes shape the integration of EPA-based assessment in clinical training. CFIR is particularly well suited to studying early implementation processes, since it enables systematic identification of contextual facilitators and barriers during the initial adoption and integration phase. As the implementation process studied is in its beginnings, we selected this framework. The CFIR categorises factors influencing implementation into five domains: Intervention characteristics, outer setting, inner setting, characteristics of the individuals involved, and implementation processes. Our approach was primarily deductive, as both the interview guide and coding framework were informed by implementation science frameworks. However, we remained attentive to data that did not align with predefined constructs, allowing inductive insights and subthemes to emerge.

### Researchers and Reflexivity

A recent medical graduate, VB had insight into the learning environment in Swiss hospitals. FS brings the perspective of a cardiologist with responsibility for postgraduate medical education in the field of cardiology. MB as senior educator and head of the Swiss Institute for Medical Education, which is in charge of residency training nationwide, provided a strategic view. EKH, offered a more external medical education researcher viewpoint.

### Setting and Sampling

This study made use of the Swiss setting, where in 2024, the cardiology training sites started using a curriculum containing EPAs. In most settings, this transition from time-based to EPA-based training meant that Mini-CEX and DOPS-based assessments were replaced by EPA-based assessments. An EPA assessment refers to a workplace-based assessment of a trainee’s performance in a specific clinical activity, based on direct observation or other clinical interactions. Supervisors provide feedback and document the trainee’s level of independence using a mobile application. We did not systematically collect data on whether participants started their training within an EPA-based curriculum or had transitioned from previous time-based assessment formats (e.g., DOPS or Mini-CEX). We selected cardiology trainees aiming for sufficient experience and exposure to EPAs. Specifically, trainees were eligible if they were currently pursuing a specialization in cardiology, had been working in a Swiss cardiology training site for at least three months, and had spent at least one month at their current training site. Furthermore, the participating hospitals were required to conduct regular EPA assessments, with a minimum of one assessment per trainee per month. Trainees also needed to have regularly assessed EPAs and be proficient in Swiss German, German, English, or Italian. Participation in this study was voluntary, and informed consent was obtained from all participants prior to data collection. A total of eight cardiology trainees participated in the study. Participant characteristics and interview details are provided in Supplementary Table 1. All interviews were conducted in German, except for one interview, which was conducted in English. Translation accuracy was reviewed by a second team member and discrepancies were resolved through discussion until consensus was reached. To ensure diversity and capture a broad range of perspectives, we included trainees from training sites which differed in hospital type and size, geographical location, the size of cardiology training cohorts, and the ratio of supervisors to trainees. We also aimed for variation in the participants’ age and gender. Sample size was guided by the concept of information power rather than a predefined number of participants [23]. Consistent with recommendations by LaDonna et al. [24], we considered data sufficiency not solely in terms of achieving saturation, but through the richness, depth, coherence, and recurrence of findings across interviews and their ability to support a credible interpretation of participants’ experiences. Our study had a narrowly focused aim and a relatively specific participant group, consisting of cardiology residents involved in the early implementation of EPAs within Swiss teaching hospitals. Data collection and analysis were conducted iteratively, using continuous comparison across interviews to identify recurring patterns as well as differing perspectives in different training contexts. Interviews were sufficiently detailed to support in-depth exploration of participants’ experiences, with interview durations ranging from 30 to 62 minutes (mean 39.5 minutes). Recruitment was stopped when additional interviews no longer contributed meaningful new insights to the developing analysis, and the dataset was considered sufficiently rich to support well-substantiated understanding of the research question.

**Figure 1:**
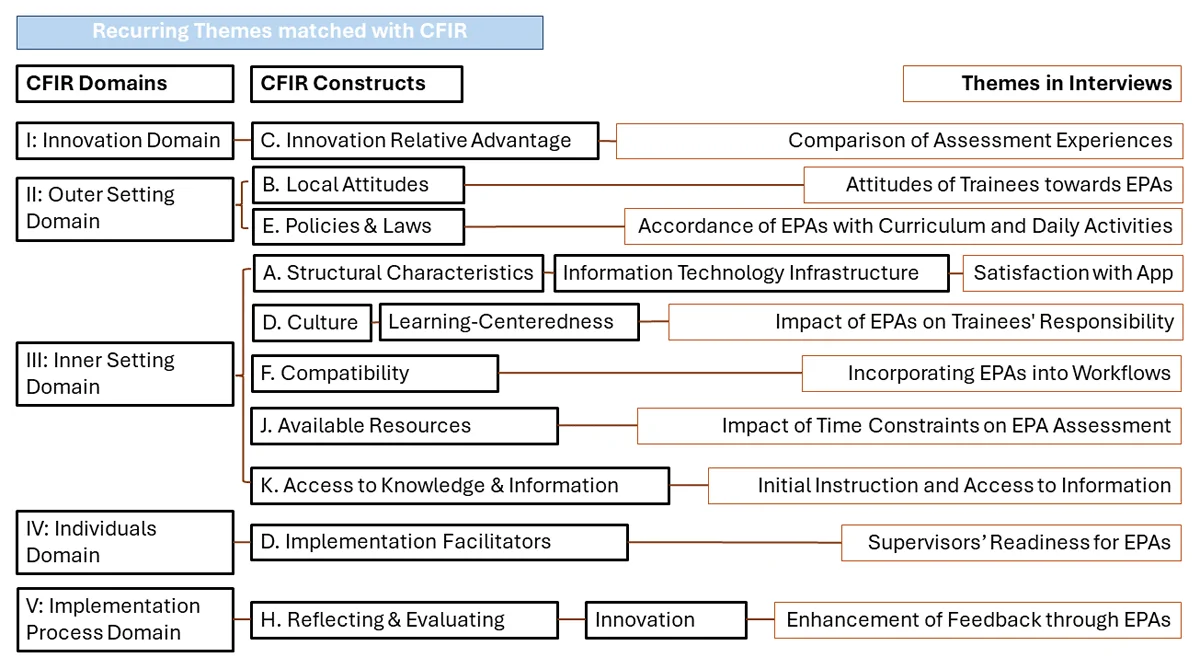
Alignment of Recurring Themes from the interviews to CFIR Domains and Constructs. *Notes:* EPA: Entrustable Professional Activity. The themes identified during the analysis of the interviews and the domains and constructs of the CFIR in comparison. CFIR constructs that were not mentioned in the interviews, are not shown.

### Ethics and Informed Consent

This study was not subject to the Swiss Human Research Act; hence the cantonal ethics committee (Kantonale Ethikkommission Bern) decided that ethical approval was not required (Req-2022-00437). Participation was voluntary. All participants gave informed consent before the study. We followed national data protection guidelines. All data were handled in accordance with applicable data protection regulations. Audio recordings and transcripts were treated confidentially and were accessible only to the research team. During transcription, all identifying information was removed and data were pseudonymised to ensure participant anonymity in reports. Electronic data were stored securely on a SIWF server for a period of ten years in accordance with cantonal data protection legislation. As some members of the research team hold roles within the Swiss medical education system, particular care was taken to minimize potential role-power dynamics. Participation was voluntary, confidentiality was ensured, and participants were informed that their responses would remain anonymous and would not influence their training or evaluation.

### Data Collection, Processing and Analysis

We sent invitation e-mails with study information and a demographic survey to trainees. Qualitative data were collected through interviews via online video conferences and transcribed using GoodTape software [25]. We applied the Framework Method, a systematic approach to analyse interviews. Meaningful units of text were identified during coding and the data was organised into a framework-based matrix to compare patterns across interviews and to identify recurring themes across interviews. This allowed for an inductive and a deductive analysis [26]. After initial coding and development of themes, findings were iteratively discussed within the research team and interpreted in relation to relevant CFIR domains and constructs. Each CFIR domain contains several constructs describing factors that may influence implementation, such as organisational culture, learning climate, available resources, and perceived advantages of an innovation. In this study, CFIR served as a sensitizing framework to inform the interview guide and support the interpretation of findings (the interview guide is provided in the Supplementary File 1). As is common in qualitative implementation studies, not all CFIR constructs were expected to be represented in the data. Data were analysed using the Framework Method. This approach uses the development of a matrix in which columns represent individual interviews and rows represent predefined codes derived from theoretical frameworks, in this case from implementation science. The resulting matrix is provided as supplementary file 2. Initial coding suggestions were developed by VB and iteratively discussed with FS. Coding decisions and interpretations were subsequently reviewed within the research team to reach consensus. Recurrent patterns and divergent cases were identified through systematic comparison within the framework matrix. Data analysis was supported by NVivo software, which was used to manage coding, organise data, and generate the framework matrix for cross-case comparison.

## Results

We identified ten themes from the analysis of the interviews, see Table 1. Our themes were interpreted in relation to relevant CFIR constructs, see Figure 1. Following this comparison, we describe and explain our ten themes as connected to the CFIR.

**Table 1:** Themes identified in the interviews, corresponding CFIR constructs, construct definitions, and study-specific interpretations.


THEME	CFIR CONSTRUCT^1^	CFIR CONSTRUCT DEFINITION^1^	INTERPRETATION IN THE CONTEXT OF THIS STUDY

1. Comparison of Assessment Experiences	Innovation Relative Advantage	The innovation is better than other available innovations or current practice.	Subjective comparison of trainees’ assessment experiences in EPA-based training with previous time-based training.

2. Trainees’ Attitudes towards EPAs	Local Attitudes	Sociocultural values (e.g., shared responsibility in helping recipients) and beliefs (e.g., convictions about the worthiness of recipients) encourage the Outer Setting to support implementation and/or delivery of the innovation.	The advantages the trainees perceive of the EPAs, what they like about them, the concerns they have and the struggles they have with their use.

3. Accordance of EPAs with Curriculum and Daily Activities	Policies & Laws	Legislation, regulations, professional group guidelines and recommendations, or accreditation standards support implementation and/or delivery of the innovation.	Whether assessments cover activities that are regularly performed and if the levels requested seem feasible. Also, whether there are assessments missing in the EPA-based curriculum.

4. Satisfaction of Trainees with App	Information Technology Infrastructure	Technological systems for tele-communication, electronic documentation, and data storage, management, reporting, and analysis support functional performance of the Inner Setting.	Handling and reception of the app.

5. Impact of EPAs on Trainees’ Responsibility	Learning-Centeredness	There are shared values, beliefs, and norms around psychological safety, continual improvement, and using data to inform practice.	Perceived influence of EPAs on trainees’ assumption of responsibility and supported autonomy in clinical practice.

6. Incorporating EPAs into workflows	Compatibility	The innovation fits with workflows, systems, and processes.	The workflows where EPAs are implemented and how shift work and different areas in the hospital are obstacles or facilitators in the implementation of EPAs.

7. Impact of Time Constraints on EPA Assessments	Available Resources	Resources are available to implement and deliver the innovation.	The obstacles the trainees face with finding enough time to assess the EPAs, the reasons behind this, the ideas on how to improve upon that and the advantages of EPAs regarding time.

8. Initial Instruction and Access to Information	Access to Knowledge & Information	Guidance and/or training is accessible to implement and deliver the innovation.	The initial instruction the trainees received, how they can access information on the EPAs and their role during the implementation.

9. Supervisors’ Readiness for EPAs	Implementation Facilitators	Individuals with subject matter expertise who assist, coach, or support implementation.	The willingness of supervisors to assess EPAs, their motivation to support implementation, and the quality of feedback they provide during EPA assessments.

10. Enhancement of Feedback through EPAs	Reflecting & Evaluating: Innovation	Collect and discuss quantitative and qualitative information about the success of the innovation.	Perceived changes in feedback frequency and quality associated with EPA-based assessments.


*Notes:* CFIR: Consolidated Framework for Implementation Research, EPA: Entrustable Professional Activity, DOPS: Direct Observation of Procedural Skills, Mini-CEX: Mini Clinical Evaluation Exercise. ^1^The CFIR constructs and their definitions are taken verbatim from the original CFIR framework [17].

### Comparison of Assessment Experiences

In the CFIR construct of ‘Innovation Relative Advantage’, we show the comparison of assessment experiences. Trainees compared their experiences with feedback and assessment in the EPA-based curriculum with previous experiences using workplace-based assessments such as Direct Observation of Procedural Skills (DOPS) and Mini Clinical Evaluation Exercises (Mini-CEXs) in time-based training. Many perceived that feedback occurred more frequently within the EPA-based system. The trainees generally preferred EPA assessments while also perceiving it as additional work. One trainee said:

‘Compared to the DOPS and Mini-CEXs, as we’re used to from internal medicine, [Authors note: To the question whether they feel that the feedback is now qualitatively or quantitatively better or more than before.] I would say yes, simply because it happens more regularly. The DOPS and Mini-CEXs are actually designed to be done, in the end, four times per contract year, but they’re often treated a bit neglectfully and without much enthusiasm, even on the side of the senior physicians. This is also because they require more effort than the EPAs, and sometimes you have to look for something specific to report on. […] With the EPAs, you already have something concrete to discuss right away. So, I think it’s significantly better.’ (Interview 3)

### Attitudes of Trainees towards EPAs

Under the domain ‘Local attitudes’, we investigated the trainees’ attitudes. While trainees generally perceived EPAs as educationally valuable and appreciated the increased opportunities for feedback described in the previous theme, many simultaneously experienced EPA assessments as an additional task in their already demanding clinical routine. Our findings indicate many trainees thought of assessing EPAs as additional work with limited perceived benefit. One trainee remarked:

Yeah, but it’s something we have to do. So we have to think about it. It’s just one more thing we have to consider. […] It’s not exactly pressure, but still one more thing.’ (Interview 5)

However, not all trainees shared this view. Some expressed a more positive attitude towards the concept of EPAs while acknowledging challenges in their current implementation:

‘I really welcome the basic idea […] but I think we still need to find the right way.’ (Interview 2)

### Accordance of EPAs with Curriculum and Daily Activities

Under the domain of ‘Policies & Laws’, we examined the accordance of EPAs with the training program and daily activities of trainees. Trainees generally reported that activities featured in the EPAs align with clinical practice. However, in smaller hospitals, rare diseases are uncommon. Some trainees worry they may be unable to complete all assessments required. One trainee remarked:

‘What we discussed at the beginning, when it was first introduced, is that […] the level required, for example, with a tricuspid stenosis, which is so rare, is something you don’t see often. And we are supposed to reach certain levels, but it’s not really realistic to achieve that. And now we are in Category A for cardiology, the highest category for training. I just wondered, how am I supposed to achieve that if there are no patients with such conditions?’ (Interview 6)

Participants described that the compatibility of EPAs varied across clinical contexts. While assessments during and after night shifts were perceived as challenging, other shifts like the late shift were described as offering better assessment opportunities. As one participant said:

‘And generally, I haven’t experienced that an evaluation with EPAs takes place after a week of night shifts. Which I find unfortunate, but that’s how it is. During late shifts, however, when a senior physician is present in the emergency department at least until 5 p.m., it works.’ (Interview 1)

In contrast, participants also reported that assessments were more difficult to conduct in settings such as outpatient clinics or during busy daytime clinical duty, where time pressure and brief supervisory encounters limited opportunities for structured feedback and EPA assessment. For example, trainees described that during busy daytime clinical duty, supervisors often had only brief contact with them, leaving little opportunity to conduct a structured EPA assessment.

### Satisfaction of Trainees with App

The theme ‘Satisfaction of Trainees with App’ refers to the ‘Information Technology Infrastructure’ in the CFIR. The trainees generally reported high satisfaction with the mobile app due to its accessibility and time efficiency. Representatively one trainee said the following:

‘I think it’s very good, yes. I find it easy to use. It’s also easy to install. […] All the senior doctors have it, so it was easily accessible to everyone. […] And I think it’s also clever. In the past, we always printed out the form and wrote on it by hand, which was time-consuming, so I actually find it very practical to implement it into everyday work.’ (Interview 2)

### Impact of EPAs on Trainees’ Responsibility

As part of ‘Learning-Centeredness’, we identified the theme of the impact of EPAs on the responsibility of trainees. The data indicate that the results of assessments of EPAs had little impact on the responsibilities trainees faced. Trainees noted that once they demonstrated certain capabilities, they were expected to assume responsibility for that task, regardless of whether an EPA had been assessed. When asked if the evaluation of their level of independence influenced the responsibility in daily activities, one trainee replied:

‘Actually, no. It’s not really like that. For example, if you’ve treated a patient with a pulmonary embolism and didn’t need much support from a senior physician, then they’re not going to stand at the bedside the next time there’s a pulmonary embolism either, unless it’s a different setting. But I think that doesn’t happen due to the EPAs, it’s actually independent of that.’ (Interview 8)

When trainees demonstrated independence during an EPA assessment, this was not automatically seen as justification for assuming full responsibility in subsequent tasks. As one trainee explained:

‘But it’s not like if you did a good job with someone’s arrhythmia, then the next time it means you have to do it completely independently, like you’re not allowed to call for help or anything. If I have a question, I can always ask.’ (Interview 2)

### Incorporating EPAs into Workflows

When looking at the construct of ‘Compatibility’, night shifts were revealed to hinder the incorporation of EPA assessments into workflows. The absence of supervisors rendered EPA assessments unfeasible:

‘So, the obstacles are the night shifts because there’s simply no one there to evaluate you, right? […] it’s difficult to catch up on what you didn’t manage during a night shift the following week when you might be back. […] after the night shifts, […] you get a few days off. And of course, during the days off, you’re not evaluated either.’ (Interview 7)

Beyond night shifts, trainees described that the feasibility of EPA assessments varied depending on the clinical context. While some settings facilitated integration into daily workflows, others posed challenges due to time pressure and limited opportunities for supervision: ‘In echocardiography it works well […], whereas in the outpatient clinic it is more difficult.’ (Interview 2)

To address these challenges, trainees suggested that more structured integration into routine practice could improve feasibility. In particular, predefined time points for assessments were perceived as helpful: ‘If there were a fixed time slot […], it would be easier.’ (Interview 1)

### Impact of Time Constraints on EPA Assessment

In the construct of ‘Available Resources’, time constraints were named as the main challenge to the EPA assessments. Trainees reported there was not enough time to add the assessments to big workload, days filled with extra hours and when prioritising more pressing activities:

‘It’s an extra task, right? I mean, we already work 50 hours a week, or we have a stressful job as it is. And then on top of that, we have to go through this as well. Of course, we appreciate feedback, but it’s an extra stress that we have to look for a senior physician with the app and say, “Give me feedback.” […] It’s an extra task.’ (Interview 4)

### Initial Instruction and Access to Information

Under the construct of ‘Access to Knowledge & Information’, it became evident that the way trainees were initially instructed on the EPA assessments and the ease of accessing related information are crucial factors. Some trainees described well-organised introductory events. Others received ad hoc instructions from colleagues. Trainees affirmed adequate access to information, but stated that supervisors could benefit from better instruction:

‘I’ve thought about whether it might be a good idea to create a short written guide for senior physicians, so they have some assistance on how to do it optimally. This way, they can do it correctly, do it more focused, and make it more goal-oriented over time.’ (Interview 6)

In some settings, trainees reported structured and comprehensive introductory sessions:

‘We had dedicated teaching sessions […] where EPAs were introduced, including live examples using the app.’ (Interview 4)

In contrast, others described a more informal and less structured approach, relying on repeated reminders rather than detailed guidance:

‘EPAs were regularly addressed during resident meetings […] and we were reminded to complete them throughout training.’ (Interview 5)

### Supervisors’ Readiness for EPAs

We identified the supervisors’ readiness for the assessments of EPA as a theme within the ‘Implementation Facilitators’ CFIR construct. Trainees reported a lack of readiness among supervisors. Supervisors who were enthusiastic about teaching, would do so without EPA assessments and the ones without interest experienced little encouragement by the new assessment tool:

‘But such feedback is independent of the EPA. If someone really likes to teach, they’ll teach regardless of whether it’s an EPA assessment or not.’ (Interview 7)

Trainees needed to take their own initiative to carry out assessments. Supervisors did not push but when specifically asked, they were willing to supervise EPAs. Trainees emphasised they would have appreciated a more proactive approach towards giving feedback formally through the use of EPAs:

‘So I always have to go to the senior physician and say, “Let’s assess an EPA now.” It has never happened that a senior physician came to me and said, “Now we need to give you written feedback.” Even when feedback is given, I have to actively ask them to do it through the EPAs.’ (Interview 6)

### Enhancement of Feedback through EPAs

In the construct of ‘Innovation’, we recognised the theme of change and potential enhancement in feedback frequency. However, trainees expressed uncertainty about the quality, ultimately stating that they did not perceive significant improvement:

‘I think we’re doing this more frequently now […]. I think you just have more opportunities to get feedback. […] So, I feel like you just have more chances to discuss things when you actively pursue it. […] Maybe one could say that the advantage of the EPAs is that you get feedback more often. And I think that’s also fundamentally our goal as trainees. For example, in one of my internal medicine trainings, I often felt like I wasn’t getting any feedback at all. And when I actually asked for it, the response was always, “Yes, you’re doing everything fine, it’s all good,” which, of course, can’t be true.’ (Interview 6)

However, trainees emphasised that the perceived benefit in feedback was not consistent and depended strongly on the individual supervisor:

‘It also depends on who is doing it [the EPA assessment] … I have the feeling the good ones [supervisors] – the good ones, that sounds so bad, so there are no good or bad ones -, but … you can tell that sadly it’s difficult to move from quantity to quality.’ (Interview 2)

## Discussion

This study explored trainees’ experiences with the early implementation of EPA-based assessment in Swiss cardiology training. While trainees generally perceived EPAs as increasing opportunities for assessment and feedback, these benefits did not automatically translate into improved feedback quality or greater responsibility in clinical practice. Interpreted through the lens of the CFIR, our findings suggest that the implementation and educational impact of EPAs depend not only on the introduction of EPA-based assessments per se but also on the interaction between structural conditions and social dynamics that shape its use in everyday clinical practice.

### Structural conditions shape implementation feasibility

Structural factors emerged as important determinants of whether EPA assessments could be integrated into routine clinical practice. Trainees primarily described challenges related to fitting assessments into existing workflows, balancing them with competing clinical demands, and ensuring adequate organisational support.

The activities included in the curriculum largely reflected routine clinical practice; however, in smaller hospitals, rare cases are uncommon. Trainees rotate across hospital types, ensuring exposure to rarer cases in larger hospitals. Overall, trainees considered the EPAs largely representative of routine cardiology practice, although some questioned the emphasis on rare clinical scenarios. This perceived alignment between EPAs and routine clinical work appeared to support implementation. Trainees highly valued the mobile app for its efficiency, especially on busy days. This finding aligns with the findings by Duggan et al. and Young & McClure who found mobile app assessments feasible and supportive of training [31,32]. The app therefore served as an important facilitator of implementation by reducing the administrative burden associated with assessments.

Trainees identified night shifts as a significant implementation barrier, as no supervisors were present to perform assessments. Teunissen et al. have already described this difficulty [33]. Trainees work autonomously during the night, so they must actively seek feedback in hindsight. These findings illustrate the challenges of integrating workplace-based assessments into clinical situations where direct observation is not routinely feasible.

Busy work schedules, prioritising more pressing activities, and extra hours hinder assessments. This finding is in line with other studies that identified time demands as key challenges [9,34], as well as implementation-focused research exploring barriers and facilitators of engagement with workplace-based assessment tools [35]. Trainees suggested allocating time slots for assessments and distributing them evenly across residency years. This approach aligns with the principles of ‘programmatic assessment’ [36]. We emphasise the allocation of time for assessments and suggest using a longitudinal approach to assessment.

Training sites organised introductory events, ranging from structured sessions to ad hoc trainee-led instructions. Many trainees reported that supervisors had received insufficient information and training regarding EPA assessments. This finding is consistent with previous research suggesting that supervisors are often expected to fulfil educational responsibilities without receiving adequate didactic training [37]. A nationwide event was held with one supervisor from each hospital. However, the subsequent dissemination of information within each institution was left to the participating supervisors. These findings suggest that successful implementation requires not only adequate implementation support for trainees but also consistent dissemination of information and training among supervisors.

Taken together, these findings suggest that implementation challenges were less related to the perceived relevance of EPAs than to the practical realities of integrating assessments into busy clinical environments. Time constraints, workflow disruptions, and inconsistent access to information may limit the extent to which increased assessment activity translates into meaningful learning opportunities, even when trainees perceive educational value in the process. Conversely, technological support, alignment with routine clinical activities, and structured implementation strategies appeared to facilitate uptake and feasibility.

### Social dynamics shape educational impact

Participants emphasised the importance of interpersonal and cultural factors. Supervisor engagement, trainee attitudes, and shared responsibility for assessment emerged as important determinants of whether EPA assessments were perceived as meaningful learning opportunities.

Many participants described being hesitant to actively request assessments from supervisors. This finding is consistent with previous research showing that trainees may be reluctant to actively seek assessment or feedback when they perceive supervisors as busy or fear negative social consequences associated with requesting evaluation [38]. Trainees experienced a tension between recognising the educational value of feedback and managing the additional effort associated with initiating and completing assessments. Although EPAs increased feedback opportunities, participants remained uncertain about whether feedback quality had improved. This observation echoes findings by Martin et al. [28] and Braund et al. [9], who described how increasing the number of assessment interactions may contribute to ‘assessment fatigue’ and reduce the educational value of feedback encounters. It is also consistent with two recent studies reporting that the quality of EPA-based feedback often lacked constructive elements [29,30]. Similarly, some trainees reported that assessments and feedback were sometimes rushed, echoing the findings by Madrazo et al., suggesting that rapid completion of EPA documentation may reduce feedback quality [27].

Supervisor engagement also emerged as an important factor influencing trainees’ experiences with EPA assessments. Thoonen et al. found that insufficiently informed supervisors may not respond adequately to trainees’ efforts to engage with EPAs, leading to frustration among trainees [39]. Participants reported that EPA assessments had little influence on the perceived level of responsibility trainees assumed in clinical practice. Many described existing supervisory relationships as sufficiently supportive, allowing them to seek assistance whenever needed regardless of their EPA entrustment level. Similarly, the finding that EPAs had limited influence on trainees’ perceived responsibility may further reflect the importance of local supervisory cultures. In settings where trainees already experience substantial autonomy combined with accessible supervision, EPA assessments may reinforce rather than fundamentally alter existing learning relationships.

The educational impact of using EPAs depends not only on the assessment process itself but also on how trainees and supervisors engage with it. Trainees valued feedback but were reluctant to initiate assessments because actively seeking assessment involved perceived social costs, including concerns about supervisors’ workload and reactions. At the same time, limited supervisor engagement was perceived to reduce the value of assessment encounters. In contrast to a recent Canadian study [40], our participants did not consider the provider of feedback to be an important factor. Consistent with earlier studies on feedback, our findings suggest that meaningful implementation requires a shared commitment to assessment and learning among both trainees and supervisors [41,42], rather than viewing EPA assessments as an administrative requirement alone.

### Interactions between structural and social factors

Synthesising the findings across the preceding sections, we suggest that successful implementation of EPA-based assessments depends on the interplay between structural conditions and social dynamics among trainees and supervisors. While trainees valued the increased frequency of feedback associated with EPAs, this benefit is contingent on sufficient time resources and effective integration into clinical workflows, such as during handovers. At the same time, supervisor preparedness and active engagement emerged as central enablers, shaping both the feasibility and perceived value of assessments. These findings highlight that EPAs do not function as a standalone intervention but rely on alignment between structural conditions (e.g., time allocation, workflow integration, digital infrastructure) and social processes (e.g., trainees’ attitudes, supervisors’ readiness, feedback culture and shared responsibility). This interpretation aligns with recent implementation-focused work arguing that successful EPA implementation depends on the work required to embed EPA practices into routine clinical care through shared understanding, stakeholder engagement, workflow integration, and adaptation to local contexts [43]. Our findings build on this perspective by illustrating that structural conditions and social dynamics jointly shape not only the feasibility of EPA implementation but also its perceived educational value for trainees. Rather than acting independently, structural conditions and social dynamics appeared to reinforce one another in shaping both the implementation and educational value of EPA assessments. When supportive structural conditions were in place, trainees and supervisors created opportunities for EPA assessments by integrating them into routine clinical practice. However, whether these opportunities translated into meaningful learning experiences depended largely on social processes, including supervisor engagement and trainees’ willingness to seek assessments, and the broader feedback culture. Even highly motivated trainees and supervisors faced difficulties when assessments competed with clinical demands or could not be integrated into existing workflows. This finding is consistent with the work of Ramani et al., who emphasised that feedback cannot be understood independently of its context and that feedback culture is shaped by the work environment [44].

The present findings highlight that the educational benefits commonly associated with EPAs depend less on the assessment tool itself than on how it is embedded within the clinical practice and learning environment. In this sense, EPAs may best be understood not as direct drivers of educational change, but as an approach that can facilitate learning when supported by favourable organisational and social conditions.

### Implications for practice and implementation

Based on the interaction between structural and social factors identified in this study, we derived practical advice from trainees’ experiences for EPA implementation. This advice focuses on supporting both the feasibility of assessment processes and their educational value. To enhance transparency, Table 2 indicates which themes informed each recommendation.

**Table 2:** Practical advice for implementing and using EPAs, derived from trainees’ experiences.


PRACTICAL ADVICE FOR EPA IMPLEMENTATION	UNDERLYING THEMES^1^

Carry out assessments carefully to ensure high-quality feedback	1, 10

Integrate assessments into existing clinical routines	3, 6, 7, 9

Allocate protected time for assessments	6, 7

Trainees should begin their assessments early during postgraduate medical education	2, 5, 8

Ensure thorough training and information for all affected parties	2, 8, 9

Improve didactic competencies of supervisors	1, 8, 9

Distribute responsibility by requiring supervisors to assess a certain number of EPAs	2, 6, 9


^1^ Theme numbers refer to the themes listed in Table 1.

### Limitations and Future Research

This study provides insight into the first steps of implementation of EPAs in cardiology training sites in Switzerland. The study focused on a single specialty within the Swiss postgraduate training context, which may limit transferability to other specialties or healthcare systems. However, this initial period can be crucial for the success of the implementation, but continuous re-evaluation will be warranted to see how the use of EPAs develops over the long term. We aimed to include trainees ranging across all years of residency, but no senior residents had enough experience with assessments of EPAs. Due to participants’ demanding schedules, interviews were relatively brief but dense in information, maintaining data depth and quality. EPA assessments currently rely on a mobile application infrastructure in Switzerland, and implementation experiences may differ in settings using other technological systems. Finally, responses may have been influenced by social desirability bias given the involvement of supervisors as stakeholders.

## Conclusion

The study explored trainees’ experiences with the early implementation of EPA-based assessments in Swiss cardiology training. While trainees perceived EPAs as increasing the frequency of feedback, this benefit did not automatically translate into improved feedback quality or changes in responsibility in clinical practice. Our findings suggest that the educational impact of EPAs depends not only on the introduction of EPA-based assessments per se, but also on how they are embedded within the structural and social context.

Successful implementation appeared to depend on the interplay between structural conditions, such as protected time, workflow integration, and organisational support, and social dynamics, including supervisor engagement, trainee attitudes, and a shared commitment to assessment and learning. Efforts to implement EPAs should therefore focus on creating the organisational and educational conditions that support meaningful learning, feedback and sustained integration into clinical practice.

## Additional Files

The additional files for this article can be found as follows:

10.5334/pme.2076.s1 Supplementary Table 1. Participant characteristics and interview details.

10.5334/pme.2076.s2 Supplementary File 1. Interview_Guide.

10.5334/pme.2076.s3 Supplementary File 2. Matrix of themes across interviews.

## Data Availability

Due to the sensitive nature of the interviews, data cannot be shared.
