# Supplementary material for: Entrustable Professional Activities in Residency Training: A Qualitative Study on Trainees’ Perceptions Using the Consolidated Framework for Implementation Research
Source: Perspect Med Educ. 2026 Nov 2;15(1):1125–37. doi: 10.5334/pme.2076 (PMC13741940; doi:10.5334/pme.2076)
Supplement: Supplementary file 1 [file pme-15-1-2076-s1.pdf]

Supplementary Table 1

Participant characteristics and interview details

Characteristics	Value
Number of participants	8
Age, mean (SD)	32.7 (4.9) years
Age range	28–43 years
Residency year, mean (SD)	3.8 (1.0)
Residency year range	2nd–5th year
Duration of cardiology training, mean (SD)	1.1 (0.9) years
Duration of cardiology training, range	4 months–3 years
Gender	5 women, 3 men
Hospital affiliation	4 university hospitals; 3 non-university tertiary care hospitals; 1 secondary care regional hospital
Interview language	7 German; 1 English
Interview duration, range	30-62 minutes
Mean interview duration (SD)	39.5 (9.9) minutes

Notes: SD = standard deviation
