# Supplementary material for: Entrustable Professional Activities in Residency Training: A Qualitative Study on Trainees’ Perceptions Using the Consolidated Framework for Implementation Research
Source: Perspect Med Educ. 2026 Nov 2;15(1):1125–37. doi: 10.5334/pme.2076 (PMC13741940; doi:10.5334/pme.2076)
Supplement: Supplementary file 2 [file pme-15-1-2076-s2.pdf]

Supplementary file 1: Interview Guide

Interview Questions for Trainees in Cardiology in EPA based Hospitals in Switzerland
**General Perception:**
• How would you describe your overall experience with the implementation of EPAs in your training program? What worked out well and what might have not worked out well?<br>• How many EPAs have you done/do you do in a month, on average?<br>• Do you generally prefer the new EPA based approach? Why, why not?<br>• What are your personal beliefs and attitudes about EPA based education?<br>• Do you need to bring initiative if you want to assess the EPAs?<br>• What do you think about the number of EPAs you have to assess?<br>• In which situation is the assessment easier, more difficult?
**Understanding and Clarity:**
• How clearly were you instructed about the expectations and requirements of the EPA framework compared to the old curriculum?<br>• Have you been informed about the reasons for the implementation?<br>• Did you receive adequate training or orientation on the EPA model before its implementation? If not, what improvements would you suggest?<br>• Which advantages do you see in EPAs? What are the reasons for the use of EPAs?<br>• How is the handling (with the app)? Are the assessments easy to find in the app?
**Responsibility:**
• Do you feel you now have an adequate amount of responsibility?<br>• Do the levels of supervision help with a gradual increase in responsibility? Do you now feel adequately prepared for the amount of independence you have in your daily activity?<br>• Are the levels of responsibility received in the EPA evaluation representative of the actual responsibility you have in the daily clinical activity?
**Skill Development:**
• How has the EPA framework impacted your ability to develop and demonstrate specific clinical skills?<br>• Do you believe that the EPA model better prepares you for real-world clinical practice? Why or why not?<br>• Do the EPAs cover the right activities?

Feedback and Assessment:
• What would you change about the EPA assessments?<br>• How do you perceive the feedback you receive under the EPA framework compared to the traditional curriculum? Why do you think that is?<br>• Are the assessment criteria for EPAs clear and fair? How do they compare to the assessments in the previous curriculum?<br>• Do you now receive more feedback?<br>• Do you receive more structured feedback?<br>• What do you consider a 'feedback'?<br>• Do you understand is as a chore that you have to actively request feedback?<br>• Are the supervisors motivated to assess your EPAs?<br>• How would you generally describe the feedback situation?
**Support and Resources:**
• What kind of support have you received from supervisors and mentors regarding the EPA implementation?<br>• How do you feel about the resources available to you for succeeding in the EPA framework (e.g., workshops, materials, mentorship)?<br>• How's the readiness of the supervisors?<br>• Is the role of the supervisors clear?<br>• Is there an allocated time slot for the assessment of EPAs?<br>• Do you have enough time for the assessments? Does it add time pressure to your daily activities?
**Challenges and Obstacles:**
• What challenges have you encountered in adapting to the use of EPAs? What challenges have you heard from other trainees? Do you think your perception is similar to what you hear from your colleagues?<br>• Are there specific aspects of the old curriculum that you feel are lacking or have been lost in the transition to EPAs?<br>• Are there any obstacles where the EPAs only work in theory but practical applications are difficult? Eg. not enough time to get the EPAs assessed
**Engagement and Motivation:**
• How has the transition to an EPA-based curriculum affected your engagement and motivation in your training?<br>• Do you feel more motivated to take ownership of your learning in the EPA framework compared to the traditional model?<br>• Do you like that the progress of your education now lies more within your hands?<br>• Do you have trust in being taught under an EPA based system? What factors impact your trust, your opinion about it?

Future Improvements:
• What suggestions do you have for improving the implementation of EPAs in other training sites? (Suggestions of things that didn't work well (negative), maybe also suggestions for things that went well, important to keep that way (positive))<br>• Which recommendations would you give to another trainee starting anew with EPAs? (Something you did well that helped you (positive), something you struggled with to be attentive about (negative))<br>• How do you envision the ideal balance between competency-based education and the traditional curriculum?<br>• Can you imagine a future in which medical education is solely based on EPAs?<br>• Would you have also already liked to do EPAs during the internships at university?
**Other:**
• Is there anything else you would like to add?
