# Supplementary material for: Entrustable Professional Activities in Residency Training: A Qualitative Study on Trainees’ Perceptions Using the Consolidated Framework for Implementation Research
Source: Perspect Med Educ. 2026 Nov 2;15(1):1125–37. doi: 10.5334/pme.2076 (PMC13741940; doi:10.5334/pme.2076)
Supplement: Supplementary file 3 [file pme-15-1-2076-s3.pdf]

Supplementary file 2: Matrix of Themes across Interviews

	1	2	3	4	5	6	7	8
Comparison between EPAs and previous evaluation tools	0	6	2	0	0	1	2	1
Attitude of trainees towards EPAs	1	6	3	1	3	2	7	3
Accordance of curriculum and daily activities	1	1	1	1	0	3	1	5
Satisfaction with app	4	2	2	5	5	4	4	5
Impact of EPAs on trainees' responsibility	1	1	0	2	1	1	2	1
Incorporating EPAs into workflows	2	2	2	1	3	1	7	1
Impact of time constraints on EPA assessment	5	1	1	6	3	7	9	5
Initial instruction and access to information	3	2	2	3	3	5	2	7
Supervisors' readiness for EPAs	3	4	2	2	6	3	9	5
Enhancement of feedback through the use of EPAs	3	7	5	3	1	3	6	4

Supplementary file 2: Matrix of themes across interviews. The matrix shows the distribution of themes across the interviews 1 to 8. The numbers per cell show how often a theme code was used in that interview transcript, e.g. the code for theme 1 was used zero times in interview A and six times in interview B. Grayscales are use to highlight the distribution, with darker shades representing a higher number of mentions of a theme and lighter shades representing a low number or no mention of a theme.
